# Meta-analysis and acupoint selection patterns of acupuncture for post-stroke foot drop

**DOI:** 10.3389/fneur.2026.1794012

**Published:** 2026-09-08

**Authors:** Yuxin Huang, Yueqi Lin, Bohui Zheng, Xinyi Zhao, Qi Su, Yina Wang, Jiaqi Ai, Wenbin Fu, Chuhua Lin

**Affiliations:** 1 Medical College of Acu-Moxi and Rehabilitation, Guangzhou University of Chinese Medicine, Guangzhou, Guangdong, China; 2 Clinical Research and Big Data Laboratory, South China Research Center for Acupuncture and Moxibustion, Medical College of Acu-Moxi and Rehabilitation, Guangzhou University of Chinese Medicine, Guangzhou, Guangdong, China; 3 The Fourth Clinical Medical College of Guangzhou University of Chinese Medicine, Shenzhen, Guangdong, China; 4 Department of Rehabilitation Medicine, The Seventh Affiliated Hospital of Sun Yat-sen University, Shenzhen, Guangdong, China; 5 The Second Clinical Medical College of Guangzhou University of Chinese Medicine, Guangzhou, Guangdong, China; 6 Department of Neurology, Dongzhimen Hospital, Beijing University of Chinese Medicine, Beijing, China; 7 The First Clinical Medical College of Guangzhou University of Chinese Medicine, Guangzhou, Guangdong, China; 8 The Second Affiliated Hospital of Guangzhou University of Chinese Medicine, Guangzhou, Guangdong, China

**Keywords:** acupuncture, data mining, foot drop, meta-analysis, stroke

## Abstract

**Background:**

Foot drop is a common complication of stroke, often causing difficulties in walking, restricted mobility, and reduced independence and quality of life. Timely systematic treatment for patients with foot drop after stroke is essential to restore foot function and improve quality of life. Acupuncture, as one of the traditional therapies in traditional Chinese medicine is widely used as an adjunctive rehabilitation intervention. However, the clinical efficacy and potential therapeutic prescriptions of acupuncture for foot drop after stroke remain unclear. Therefore, the purpose of this study was to evaluate the effects of acupuncture-based interventions for post-stroke foot drop and characterize acupoint selection patterns.

**Methods:**

We searched eight databases, namely, Embase, PubMed, Web of Science, Cochrane Library, Wanfang Database, China National Knowledge Infrastructure, VIP Database, and China Biology Medicine Database, from inception to January 2025. Randomized controlled trials evaluating acupuncture-based interventions for post-stroke foot drop were included. Studies were included or excluded based on prespecified eligibility criteria. The included studies were assessed for quality using the Cochrane Risk of Bias tool, and certainty of evidence was assessed using GRADE. The primary outcome measure was the Fugl–Meyer Assessment (FMA). Secondary outcomes included clinical effective rate, active range of motion (AROM), tibialis anterior electromyography (EMG), Barthel Index (BI), Berg Balance Scale (BBS), Modified Ashworth Scale (MAS), triceps surae muscle tone (TSMT), Functional Ambulation Category (FAC), and co-contraction ratio during ankle dorsiflexion (CR). Meta-analysis was performed using RevMan V5.3 software, and acupoint selection patterns were analyzed using IBM SPSS Statistics 18.0 and 28.0. To address clinical differences among interventions, studies were analyzed separately as traditional acupuncture, electroacupuncture, and special acupuncture techniques.

**Results:**

A total of 59 relevant studies were included, with 4,596 participants; 2,290 received conventional treatment alone, and 2,306 received acupuncture-based interventions in addition to conventional treatment. To reduce clinical heterogeneity associated with different acupuncture modalities, studies were stratified into traditional acupuncture, electroacupuncture, and special acupuncture subgroups for quantitative synthesis. Traditional acupuncture and electroacupuncture were both associated with improved FMA scores compared with conventional treatment alone [mean difference (MD) = 6.25, 95% confidence interval (CI) 5.11–7.40, *p* < 0.00001; MD = 7.99, 95% CI 6.05–9.93, *p* < 0.00001, respectively]. Favorable effects were also observed for AROM, MAS, BBS, BI, and other functional outcomes in modality-specific analyses. Traditional acupuncture was associated with improved EMG and FAC scores and reduced TSMT and CR, whereas evidence for special acupuncture techniques was limited for most outcomes. The three most frequently used acupoints were Yanglingquan (GB34; 37 times, 9.37%), Zusanli (ST36; 37 times, 9.37%), and Jiexi (ST41; 31 times, 7.85%). Substantial heterogeneity was observed for several outcomes, and the certainty of evidence ranged from moderate to low. Exploratory publication bias assessments suggested possible small-study effects for selected outcomes; notably, the adjusted estimate for clinical effective rate crossed the line of no effect. Adverse events were insufficiently reported to permit a reliable assessment of safety.

**Conclusion:**

Traditional acupuncture and electroacupuncture may provide additional functional benefits when used alongside conventional treatment for post-stroke foot drop. However, substantial heterogeneity, predominantly unclear or high risk of bias, possible small-study effects, and moderate-to-low certainty of evidence limit confidence in the precise magnitude of benefit and preclude conclusions regarding the superiority of specific acupuncture modalities or prescriptions. Larger, rigorously designed, and transparently reported randomized controlled trials are needed to confirm efficacy, clarify comparative effectiveness, and adequately assess safety.

**Systematic review registration:**

The PROSPERO registration number is CRD42024520076.

## 1 Introduction

Stroke is one of the most common acute cerebral vascular diseases, which has a great impact on the health and the economy. It caused approximately 29,616 deaths and 947,562 disability-adjusted life years (DALYs) due to ischemic stroke worldwide in 2021 alone (1). The Global Burden of Disease study indicates that the lifetime risk of stroke in the Chinese population is as high as 39.3%, the highest in the world (2). In recent years, the rapid aging of China’s population, combined with the increasing pressures of modern society and changes in dietary habits, has led to a rising incidence of stroke, posing a serious threat to the health and safety of the elderly (3). When a stroke occurs, the brain tissue becomes ischemic and hypoxic due to insufficient blood supply, resulting in damage and gradual loss of functions such as movement, sensation, cognition, and emotion. If not treated promptly during the acute phase, it can lead to severe sequelae that seriously endanger human health. Foot drop is a common complication of stroke. Research has shown that over 54% of stroke patients experience it after treatment (4, 5). Following a stroke, patients experience impaired brain function and assume a position dominated by lower-level central reflexes. Immobility further alters the activity characteristics of local muscles, tendons, and surrounding connective tissues, leading to muscle stiffness, increased muscle tone, and exacerbated joint movement disorders. This leads to problems with the dorsiflexion of the ankle and the impairment of the lower limbs, such as standing, walking, and balance (6), which has a significant impact on patients’ daily activities and quality of life (7).

Currently, the main treatments for foot drop after stroke include conventional therapy and rehabilitation training. Conventional therapy involves the use of medications to nourish brain neurons, maintain electrolyte balance, regulate lipid levels, stabilize atherosclerotic plaques, and reduce inflammation and swelling (8). Alternatively, etiological treatment with muscle relaxants or antispasmodic drugs may be used, although their therapeutic effects are limited and may cause adverse reactions such as gastrointestinal discomfort, headache, and dizziness in some patients. Rehabilitation training aims to improve muscle strength, flexibility, balance, and gait and is the primary method for treating foot drop after stroke in China. However, its overall efficacy is not satisfactory (9). Numerous clinical studies have shown that acupuncture combined with rehabilitation training can improve the problem of lower limb dysfunction in patients after stroke (10, 11). As one of the most important methods in Traditional Chinese Medicine, acupuncture is characterized by its safety, convenience, low cost, significant efficacy, and minimal adverse reactions, making it valuable for clinical application and promotion. In recent years, the number of clinical trials on acupuncture for foot drop after stroke has increased. Many research studies have shown that acupuncture can reconstruct brain function after injury, and it can efficiently improve the dysfunction of limbs (12).

Although acupuncture has exhibited potential value in the clinical management of foot drop after stroke, existing studies have notable limitations in demonstrating its clinical efficacy. Specifically, the core evidence gaps regarding its overall therapeutic effect, safety, and consistency of outcomes have not been systematically clarified, and in-depth comparative analyses of the intervention differences among various acupoint prescriptions are lacking. Moreover, a unified conclusion has not yet been formed on the screening and verification of relevant potential treatment regimens. Current meta-analyses have mostly focused on the intervention effects of electrical stimulation therapies for foot drop after stroke. Relevant research has included electroacupuncture in the category of electrical stimulation therapies for comparative analysis and confirmed its advantages in improving patients’ ankle dorsiflexion function and lower limb motor function; however, it has not conducted special investigations on the acupoint compatibility patterns and prescription rules of acupuncture itself (13). To date, there have been no dedicated meta-analyses on acupuncture for foot drop after stroke, making it difficult to provide targeted and scientific clinical references in terms of acupoint prescription optimization and efficacy specificity verification. Therefore, it is imperative to conduct a special meta-analysis to systematically explore the efficacy and safety of acupuncture in the treatment of foot drop after stroke and clarify the intervention characteristics of different acupoint prescriptions. This study intends to analyze the above core dimensions, aiming to provide evidence-based references for the clinical acupuncture treatment of this condition.

## 2 Methods

After determining the research topic, we registered our study on PROSPERO (14) and obtained the registration number CRD42024520076. This systematic review and meta-analysis was conducted and reported in accordance with the Preferred Reporting Items for Systematic Reviews and Meta-Analyses (PRISMA) 2020 guidelines.

### 2.1 Literature search

#### 2.1.1 Search strategy

We conducted a comprehensive search of the eight databases from their establishment to January 2025. The databases included Embase, PubMed, Web of Science, the Cochrane Library, Wanfang Database, China National Knowledge Infrastructure (CNKI), VIP Database, and China Biology Medicine Database (CBM). The search terms were composed of three parts: “post-stroke,” “foot drop,” and “acupuncture treatment.” Specifically, the “post-stroke” terms included stroke, post-stroke, cerebral infarction, cerebral ischemia, cerebral hemorrhage, and cerebrovascular accident; the term for “foot drop” was “foot drop”; and the “acupuncture treatment” terms included acupuncture, warm acupuncture, electroacupuncture, fire needle, plum blossom needle, needle-knife, filiform needle, scalp acupuncture, and auricular acupuncture. The detailed search strategy is provided in Supplementary Material 1 (Search Strategy).

### 2.2 Inclusion and exclusion criteria

#### 2.2.1 Study type

We included only randomized controlled trials (RCTs) that focused on acupuncture treatment for foot drop after stroke. All included studies were published in peer-reviewed journals or databases without involving personal privacy.

#### 2.2.2 Inclusion criteria

The inclusion criteria were as follows:

1. Patients with stroke who meet the diagnostic criteria of the Chinese Guidelines for the Diagnosis and Treatment of Cerebral Hemorrhage (2019) (15) or the Chinese Guidelines for the Diagnosis and Treatment of Acute Ischemic Stroke (2018) (16) were included: Among them, cerebral hemorrhage must satisfy “acute onset, accompanied by headache, vomiting, disturbance of consciousness or focal neurological deficits, intracerebral hemorrhage foci confirmed by cranial computed tomography (CT) or magnetic resonance imaging (MRI), and exclusion of traumatic cerebral hemorrhage and secondary etiologies”; acute ischemic stroke must satisfy “acute onset, manifested as focal neurological deficits, exclusion of cerebral hemorrhage by cranial CT, clear ischemic location confirmed by MRI diffusion-weighted imaging, and exclusion of non-vascular cerebral etiologies.” Meanwhile, the diagnosis of foot drop after stroke must meet the following criteria: occurrence of unilateral or bilateral ankle dorsiflexion dysfunction after stroke onset, accompanied by toe flexion and step page gait during walking; physical examination shows ankle dorsiflexion muscle strength ≤ grade 3; foot drop is confirmed to be caused by stroke lesions through clinical symptoms, neurological examination, and imaging examinations (cranial CT/MRI); foot drop caused by other factors such as peripheral neuropathy, muscle diseases, and osteoarticular injuries is excluded.
2. Patients who, while seated with legs naturally hanging, were unable to actively dorsiflex or invert/evert the foot; had a score of less than 2 on the Modified Ashworth Scale (MAS); and were in Brunnstrom Stage > III.
3. No restrictions on race, nationality, age, or gender.

#### 2.2.3 Exclusion criteria

The inclusion criteria were as follows:

1. Foot drop occurring before the stroke.
2. Patients with cognitive impairment who could not cooperate with the treatment.
3. Research studies in which the intervention is applied to both the control and experimental groups involved acupuncture combined with multiple other therapies, and these interventions were not used in the same manner or dosage in both groups.
4. Studies focusing on the selection of acupuncture points, implementation of acupuncture techniques, or the effects of stimulating specific sites (e.g., myofascial trigger points) on therapeutic efficacy.

#### 2.2.4 Interventions

All eligible acupuncture-based interventions identified in the included randomized controlled trials were retained; no acupuncture modality was selected on the basis of its presumed efficacy. For analytical purposes, interventions were classified according to their principal mode of stimulation and treatment delivery. Traditional acupuncture referred to manual needle insertion and manipulation without electrical stimulation. Electroacupuncture was classified separately because it delivers needle stimulation through an externally applied electrical current, with distinct stimulation parameters such as frequency and intensity. Special acupuncture techniques included approaches such as scalp acupuncture, fire needle, and intradermal needle, which differ from conventional manual needling in stimulation mode, anatomical target, thermal stimulation, or needle retention procedure. This operational classification was used to avoid inappropriate pooling of clinically distinct interventions, rather than to imply that the three categories were mechanistically homogeneous or to determine the superiority of any acupuncture modality. Because the special acupuncture category included relatively few and procedurally diverse studies, its findings were interpreted as exploratory, and outcomes reported by only one study were summarized descriptively. The control groups received conventional rehabilitation training, pharmacological treatment, or routine care.

#### 2.2.5 Outcome measures

All outcome measures from the included studies were collected and ranked. Based on the recommendations from the “Chinese Guidelines for the Diagnosis and Treatment of Cerebral Hemorrhage (2019)” and “Chinese Guidelines for the Diagnosis and Treatment of Acute Ischemic Stroke (2018),” we selected the top 10 most frequently reported outcome measures after excluding those with significant calculation differences across studies:

##### 2.2.5.1 Primary outcome

The primary outcome included the following: Fugl-Meyer Assessment Scale (FMA) to evaluate overall motor function.

##### 2.2.5.2 Secondary outcomes

Secondary outcomes included the following:

1. Clinical effective rate.
2. Active range of motion (AROM) to evaluate ankle joint mobility.
3. Electromyogram (EMG) of the tibialis anterior to assess muscle activity.
4. Barthel Index (BI) of activities of daily living (ADL) to assess daily living abilities.
5. Berg Balance Scale (BBS) to evaluate overall balance.
6. Modified Ashworth Scale (MAS) to assess lower limb muscle spasticity.
7. Triceps surae muscle tone.
8. Holden Functional Ambulation Category Scale (FAC) to evaluate walking ability.
9. Co-contraction ratio (CR) of the limbs in ankle dorsiflexion to assess muscle contraction ability.

### 2.3 Data extraction

Data on the source of the literature, author names, acupuncture prescriptions, and acupuncture methods were entered into Excel 2019. The names, locations, and meridians of acupuncture points were referenced from “Acupuncture and Meridian Studies.” Two researchers (LYQ and ZXY) independently screened the retrieved literature according to the inclusion and exclusion criteria and cross-checked the results. Any disagreements were resolved through team discussion and consultation with a third researcher (HYX). Missing data were obtained by contacting the corresponding authors of the articles. Extracted data included outcome measures, the first author’s name, publication date, intervention measures, intervention duration, and results. If different studies defined an outcome measure differently, the two researchers carefully reviewed the definitions, consulted relevant materials, and decided whether the measures could be combined. If they could not reach a consensus, a third researcher was consulted.

### 2.4 Quality assessment of literature

Two independent researchers used Note Express software to manage and screen the literature. They screened the literature and extracted data by reading the titles, abstracts, and full texts and then compared and verified the results. Any divergence was worked out by a third party. According to the items for assessing the risk of bias provided by Cochrane (17), the two researchers (LYQ and HYX) assessed each included study in the following six domains:

1. Whether the researchers could predict randomization and allocation outcomes.
2. Whether participants were unaware of their group assignment; whether evaluators were blinded to the group being assessed.
3. Whether the outcomes could not be predicted.
4. Whether all reported outcomes in the article had specific results.
5. Whether all possible outcomes of the study were reported.
6. Whether there were other potential biases, such as defective experimental design.

If there is a disagreement within the assessment team, the third researcher will step in to negotiate and handle the situation.

### 2.5 Data analysis

#### 2.5.1 Meta-analysis

For the meta-analysis, we used RevMan software (Version 5.3) to perform statistical analyses, including bias risk assessment plots, forest plots for each outcome measure, and funnel plots to evaluate publication bias. If significant heterogeneity was observed among the studies, we conducted sensitivity and subgroup analyses.

##### 2.5.1.1 Analysis of individual studies

For dichotomous outcomes, risk ratio (RR) with 95% confidence interval (CI) was calculated. For continuous outcomes measured using the same scale and unit across studies, mean differences (MD) with 95% CI were calculated. Standardized mean difference (SMD) with 95% CI was planned for outcomes assessing the same construct using different measurement scales or units, if applicable. All effect estimates were presented with 95% CI.

##### 2.5.1.2 Heterogeneity analysis

After the initial analysis, we assessed heterogeneity among the studies using *I^2^* values and the chi-squared tests. When *I*^2^ ≤ 50% and *p* ≥ 0.05, we considered the heterogeneity to be insignificant (6, 77) and used a fixed-effect model for analysis. When *I*^2^ > 50% and *p* < 0.05, we considered significant heterogeneity to be present and used a random-effect model. To explicit the origin of heterogeneity, we explored the research method, intervention duration, patient gender, age, and other covariates on effect size through sensitivity and subgroup analyses until the meta-analysis was deemed rigorous and complete. If a study could not be excluded, we used a random-effect model for pooling.

##### 2.5.1.3 Sensitivity analyses

Sensitivity analyses were conducted to assess the robustness of the pooled estimates. For meta-analyses including at least three studies, leave-one-out analyses were performed by sequentially removing each study and recalculating the pooled effect estimate, 95% CI, and *I^2^* statistic.

A study was considered potentially influential when its exclusion materially changed the magnitude or direction of the pooled effect, the statistical significance of the result, or the extent of heterogeneity. Studies were not excluded from the primary analysis solely because their removal reduced heterogeneity. Instead, potential sources of influence were explored by examining differences in acupuncture protocol, treatment duration, participant characteristics, control interventions, and the use of unmatched co-interventions. Additional sensitivity analyses were conducted by excluding studies in which background treatments differed substantially between the intervention and control groups, where applicable.

##### 2.5.1.4 Publication bias analysis

Potential risk of bias due to missing evidence was assessed only for modality-specific meta-analyses that included at least 10 studies and showed sufficient variation in study precision. Funnel plots were visually inspected for asymmetry, and Egger’s regression test was used to evaluate potential small-study effects where appropriate.

Evidence of funnel plot asymmetry was interpreted cautiously because asymmetry may arise from publication bias, selective outcome reporting, methodological limitations of smaller studies, or genuine clinical heterogeneity. Therefore, funnel plot asymmetry was not considered diagnostic of publication bias. Although potential small-study effects were identified, trim-and-fill analyses were conducted as exploratory sensitivity analyses to examine the potential influence of missing studies on the pooled estimates.

#### 2.5.2 GRADE assessment

The certainty of evidence for each outcome was assessed using the Grading of Recommendations Assessment, Development and Evaluation (GRADE) approach. Since all included studies were randomized controlled trials, the initial certainty of evidence for each outcome was rated as high. The certainty of evidence was then downgraded according to five domains: risk of bias, inconsistency, indirectness, imprecision, and publication bias.

Risk of bias was judged based on the Cochrane Risk of Bias assessment of the included studies. Inconsistency was evaluated according to the magnitude of heterogeneity, mainly using the *I^2^* statistic, together with the results of subgroup and sensitivity analyses. Indirectness was assessed by considering whether the participants, interventions, comparisons, and outcomes were consistent with the clinical question of acupuncture for post-stroke foot drop. Imprecision was evaluated according to the width of the 95% CI, whether the CI crossed the line of no effect, and the sample size of each outcome. Publication bias was assessed using funnel plots when more than 10 studies were included for an outcome; when fewer than 10 studies were available, publication bias was considered not reliably assessable.

The final certainty of evidence was classified as high, moderate, low, or very low. The GRADE assessment was performed for the following 10 outcomes: FMA, clinical effective rate, AROM, EMG, BI, BBS, MAS, triceps surae muscle tone, FAC, and CR.

#### 2.5.3 Data mining

For data mining, we used SPSS 28.0 statistical software to perform frequency analysis, hierarchical clustering, and dendrogram plotting. We also used SPSS 18.0 to conduct association rule analysis and create association network diagrams using the Apriori algorithm.

## 3 Results

### 3.1 Meta-analysis

#### 3.1.1 Literature screening

**Figure 1:**
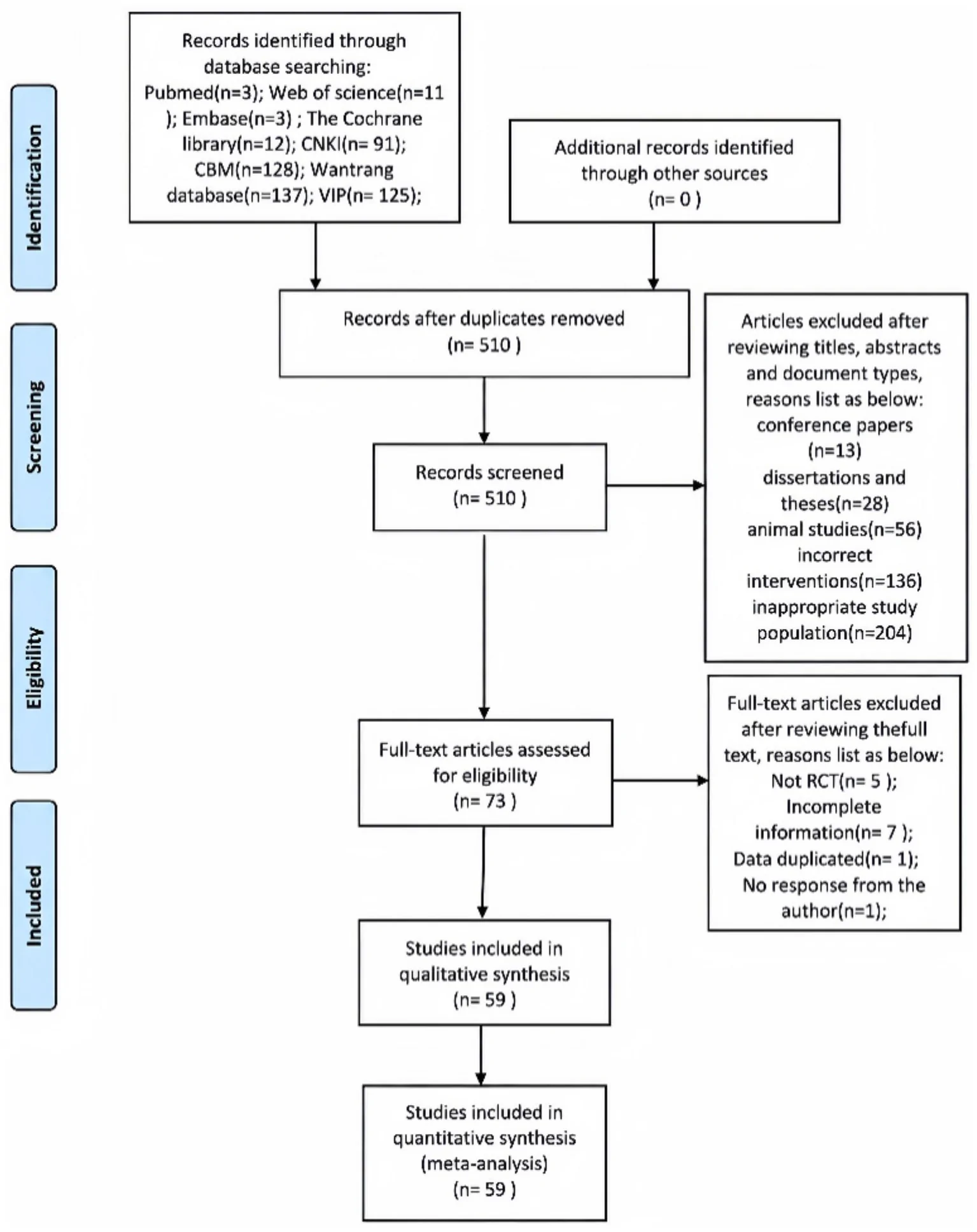
PRISMA flow diagram.

A total of 510 relevant studies were identified through the literature search. After a rigorous systematic screening process, 59 eligible studies were finally brought into the analysis (3, 8, 10, 18–73). The circumstantial screening process is demonstrated in Figure 1. Data characteristics of these 59 studies are presented in Supplementary Material 2.

#### 3.1.2 Characteristics of included studies

The 59 included literature studies (3, 8, 10, 18–73) were all put into effect in China, with a total sample size of 4,596 participants. Specifically, 2,290 participants received conventional treatment alone, while 2,306 participants received a combination of conventional treatment and acupuncture therapy.

#### 3.1.3 Risk of bias assessment

**Figure 2:**
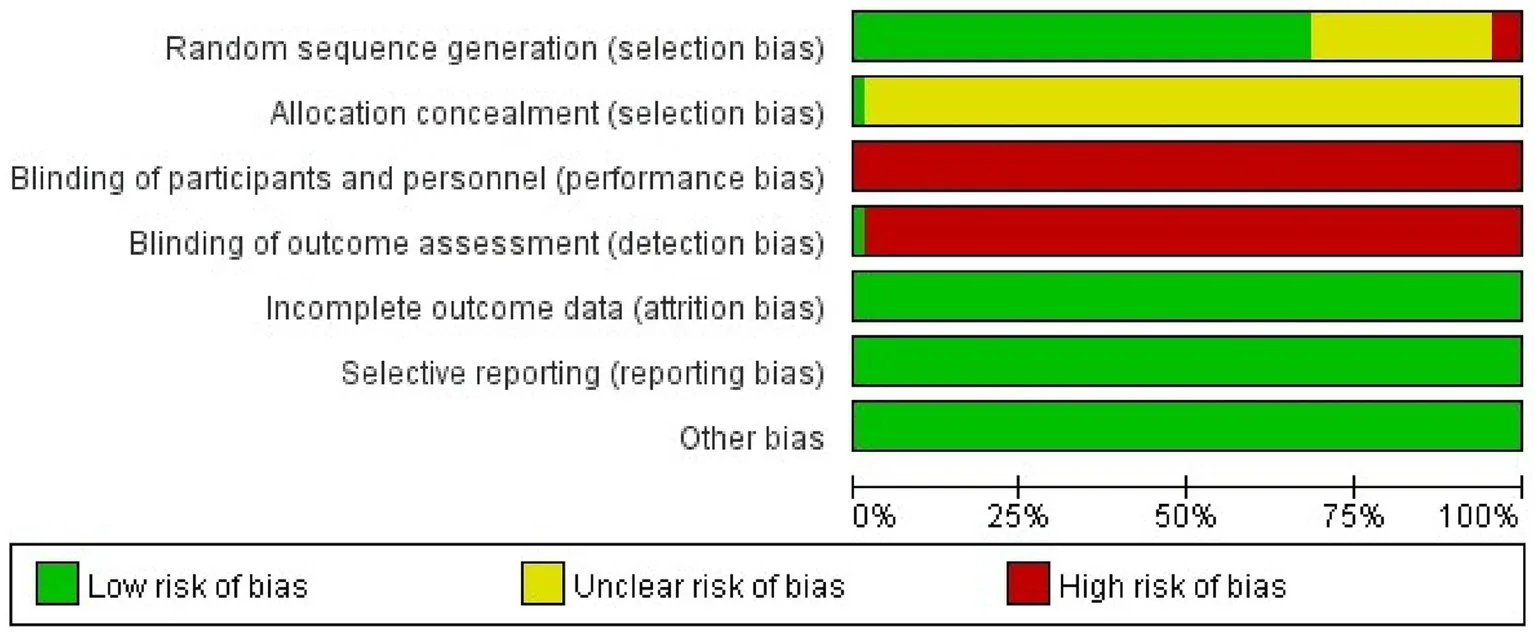
Quality map of included literature.

Among the included studies, the risk of bias in random sequence generation was unclear in 16 studies (3, 21, 24, 25, 27, 32, 41, 45, 49–51, 56, 61, 64, 68, 72) and high in 3 studies (35, 54, 67). The risk of bias in allocation concealment was unclear in 58 studies (3, 8, 10, 18–60, 62–73). The risk of bias related to blinding of participants and personnel was high in all 59 studies (3, 8, 10, 18–73). The risk of bias related to blinding of outcome assessors was high in 58 studies (3, 8, 10, 18–30, 32–73). These assessments are illustrated in Figure 2 and Supplementary Material 3.

#### 3.1.4 Stratified meta-analysis results by acupuncture type

**Figure 3:**
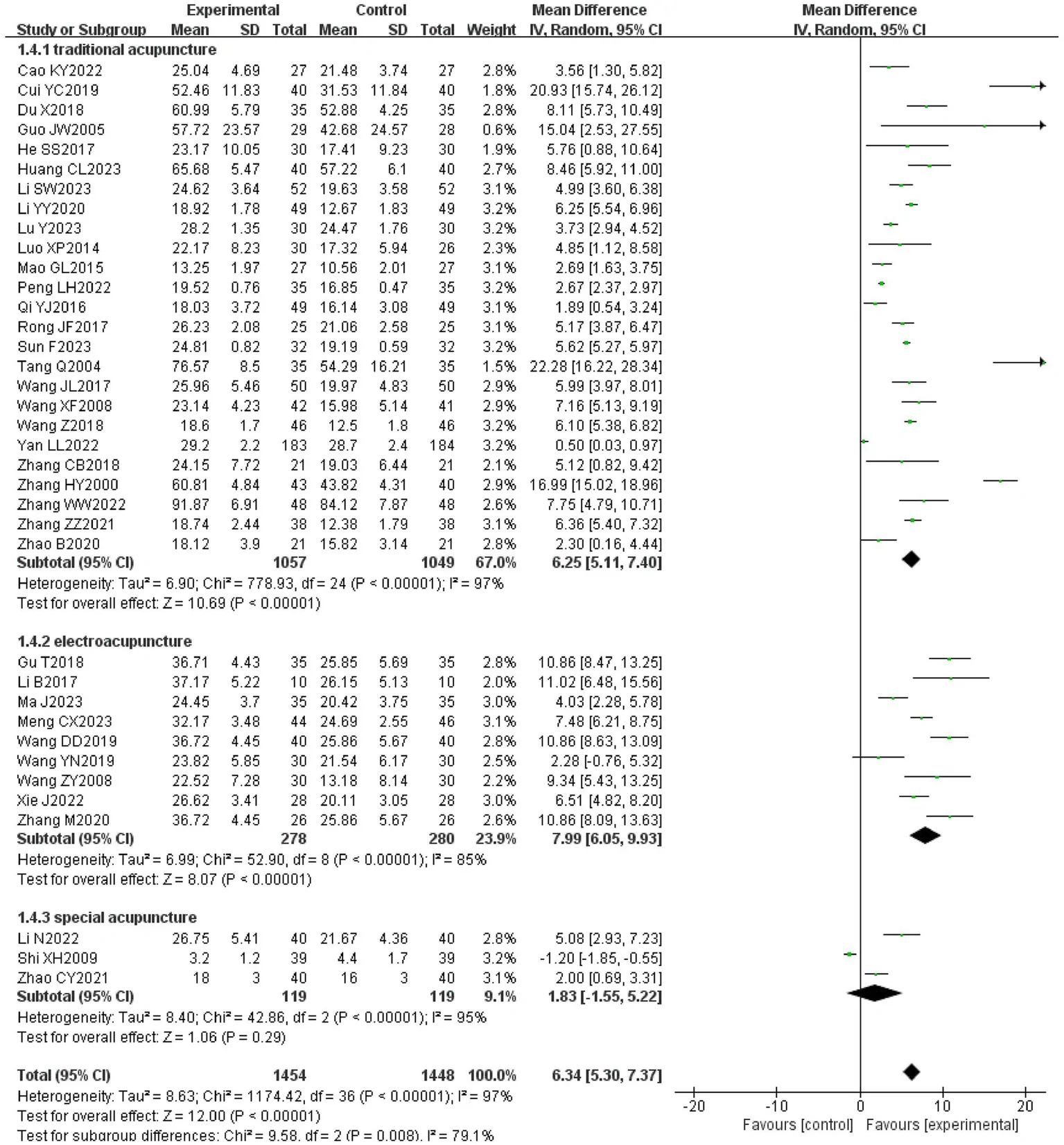
Forest diagram of FMA.

**Figure 4:**
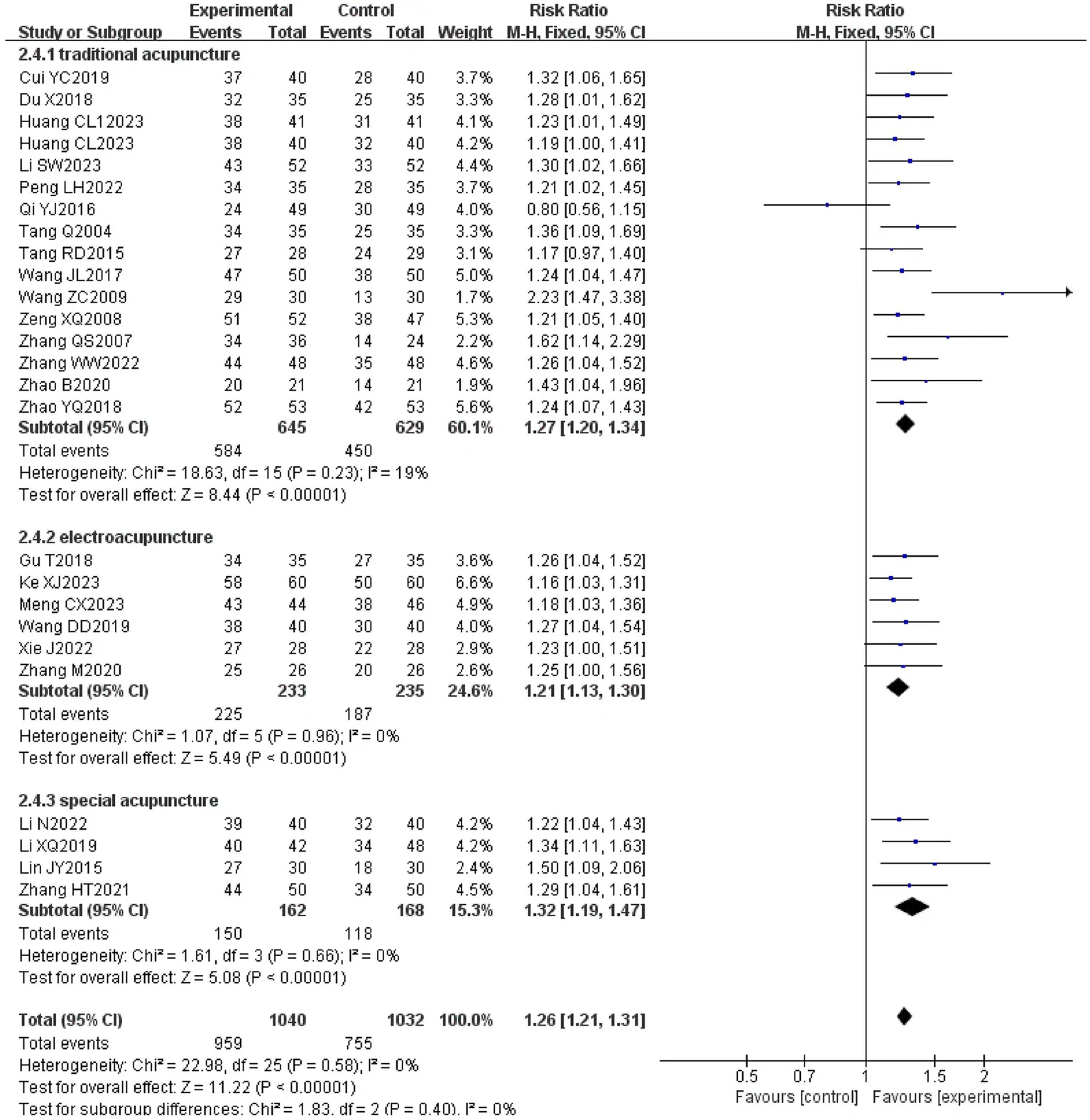
Forest diagram of clinical effective rate.

**Figure 5:**
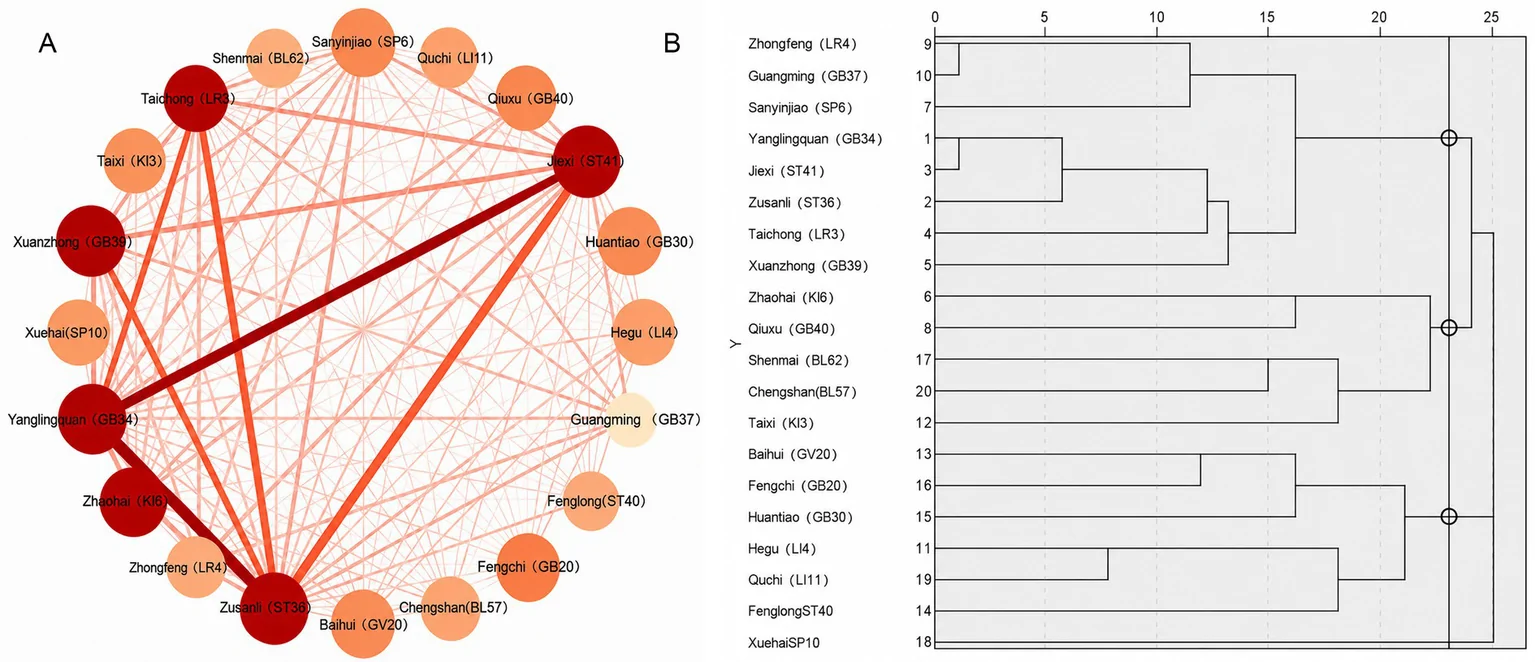
Association network and hierarchical clustering of high-frequency acupoints for post-stroke foot drop (left: association network of high-frequency acupoints. Node size and color intensity represent the frequency of acupoint use, whereas line thickness and color intensity represent the strength of association between acupoints. Right: hierarchical clustering dendrogram of high-frequency acupoints based on their co-occurrence patterns in the included studies).

Given the material differences among acupuncture modalities in stimulation procedures, treatment protocols, and potential mechanisms, studies were stratified into traditional acupuncture, electroacupuncture, and special acupuncture groups before quantitative synthesis to avoid inappropriate pooling across clinically distinct interventions. Separate meta-analyses were conducted within each subgroup when at least two clinically comparable studies were available; outcomes reported by only one study within a subgroup were summarized descriptively. Heterogeneity within each subgroup was assessed using the *I^2^* statistic. Substantial residual heterogeneity remained in several analyses and may reflect differences in stroke severity and stage, treatment frequency and duration, acupoint selection, background rehabilitation, co-interventions, and outcome assessment timing. Therefore, pooled estimates with substantial heterogeneity were interpreted cautiously. Forest plots for all outcomes are presented in Figures 3–5.

##### 3.1.4.1 FMA

A total of 37 studies (8, 10, 18–20, 22, 23, 25, 28, 29, 32, 33, 35, 37, 39, 40, 42, 43, 45–48, 51, 53, 54, 56, 58, 59, 61–65, 67–69, 73) reported the FMA outcomes. In the traditional acupuncture subgroup, 25 studies were included. Substantial heterogeneity was observed (*I^2^* = 97%, *p* < 0.00001); therefore, a random-effects model was used. The pooled analysis demonstrated a significant improvement in FMA scores compared with conventional treatment [MD = 6.25, 95% CI (5.11, 7.40), *p* < 0.00001]. Similarly, nine studies were included in the electroacupuncture subgroup, in which substantial heterogeneity was also observed (*I^2^* = 85%, *p* < 0.00001). A random-effects model was used, and electroacupuncture was associated with higher FMA scores than the control intervention [MD = 7.99, 95% CI (6.05, 9.93), *p* < 0.00001]. In the special acupuncture subgroup included in the three studies, considerable heterogeneity was also detected (*I^2^* = 95%, *p* < 0.00001). However, the pooled effect was not statistically significant [MD = 1.83, 95% CI (−1.55, 5.22), *p* = 0.29]. Overall, significant differences were identified among acupuncture modalities (*p* = 0.008, *I^2^* = 79.1%). These findings suggest that both traditional acupuncture and electroacupuncture were superior to conventional treatment in improving motor function after stroke, with electroacupuncture demonstrating the greatest improvement in FMA scores.

##### 3.1.4.2 Clinical effective rate

A total of 26 studies (3, 8, 19, 22, 23, 28–30, 35, 37, 38, 40, 41, 48–52, 59, 63, 64, 66, 69–71, 73) reported the clinical effective rate outcomes. The traditional acupuncture subgroup included 16 studies and demonstrated low heterogeneity among the included studies (*I^2^* = 19%, *p* = 0.23). Therefore, a fixed-effects model was applied. The pooled analysis showed a statistically significant improvement compared with conventional treatment [RR = 1.27, 95% CI (1.20, 1.34), *p* < 0.00001]. The electroacupuncture subgroup included six studies and demonstrated good homogeneity (*I^2^* = 0%, *p* = 0.96). A fixed-effects model revealed a statistically significant benefit in favor of electroacupuncture [RR = 1.21, 95% CI (1.13, 1.30), *p* < 0.00001]. The special acupuncture subgroup included four studies and also showed no heterogeneity (*I^2^* = 0%, *p* = 0.66). The pooled analysis demonstrated a statistically significant improvement compared with conventional treatment [RR = 1.32, 95% CI (1.19, 1.47), *p* < 0.00001]. Overall, no significant differences were observed among acupuncture modalities (*p* = 0.40, *I^2^* = 0%), indicating that all three acupuncture modalities were superior to conventional treatment in improving clinical effective rate.

##### 3.1.4.3 AROM

A total of 15 studies (21, 25, 31–33, 35, 39, 40, 42, 43, 55, 57, 60, 62, 63) reported the AROM outcomes. The traditional acupuncture subgroup included seven studies and demonstrated substantial heterogeneity among the included studies (*I^2^* = 83%, *p* < 0.00001). Sensitivity analysis did not reduce the heterogeneity, so a random-effects model was employed for the meta-analysis. The pooled analysis showed a statistically significant improvement in AROM compared with conventional treatment [MD = 3.71, 95% CI (2.63, 4.79), *p* < 0.00001]. The electroacupuncture subgroup included six studies and also showed considerable heterogeneity (*I^2^* = 94%, *p* < 0.00001). Therefore, a random-effects model was used. The pooled effect size demonstrated a statistically significant benefit in favor of electroacupuncture [MD = 2.50, 95% CI (0.84, 4.16), *p* = 0.003]. The special acupuncture subgroup included two studies and showed no significant heterogeneity (*I^2^* = 0%, *p* = 0.51). The pooled analysis demonstrated a statistically significant improvement in AROM compared with conventional treatment [MD = 5.07, 95% CI (4.27, 5.86), *p* < 0.00001] (Supplementary Figure S1). However, this estimate was based on only two studies and should be interpreted cautiously. The absence of heterogeneity suggests a high degree of consistency between the included studies. However, this finding should be interpreted with caution due to the limited number of studies available. Overall, significant differences were identified among acupuncture modalities (*p* = 0.010, *I^2^* = 78.3%), indicating that the magnitude of treatment effect varied according to acupuncture type. These findings suggest that traditional acupuncture, electroacupuncture, and special acupuncture were all superior to conventional treatment in improving AROM. Although the pooled estimate was numerically larger in the special acupuncture subgroup, this finding was based on only two studies and should not be interpreted as evidence of superiority over other acupuncture modalities.

##### 3.1.4.4 EMG

A total of 16 studies (3, 10, 30, 32, 33, 39, 40, 43, 50, 55, 63–66, 69, 73) reported the EMG outcomes. According to the predefined classification of acupuncture modalities, the included studies were divided into traditional acupuncture and electroacupuncture subgroups for analysis. Although one study by Zhang et al. (50) involving special acupuncture and moxibustion reported the results of EMG, only one study data could not complete the heterogeneity analysis, so the quantitative comprehensive analysis of special acupuncture and moxibustion subgroups was not carried out. Therefore, only 15 articles were included for analysis after exclusion.

The traditional acupuncture subgroup comprised 12 studies. Considerable heterogeneity was observed across the included studies (*I^2^* = 98%, *p* < 0.00001), and a random-effects model was therefore adopted. Meta-analysis demonstrated that traditional acupuncture was associated with higher EMG values than the control intervention [MD = 17.92, 95% CI (12.95, 22.89), *p* < 0.00001]. Three studies were included in the electroacupuncture subgroup. The heterogeneity among studies was low to moderate (*I^2^* = 31%, *p* = 0.23), indicating relatively good consistency across the included trials. A random-effects model showed that electroacupuncture was not associated with a statistically significant difference in EMG values compared with the control intervention [MD = 4.71, 95% CI (−0.56, 9.99), *p* = 0.08] (Supplementary Figure S2). Sensitivity analysis in the Supplementary Figure S3 identified the study by Wang et al. (33) as a major source of heterogeneity. After excluding this study, heterogeneity was completely eliminated (*I^2^* = 0%, *p* = 0.44), and the pooled effect became statistically significant [MD = 3.12, 95% CI (2.42, 3.82), *p* < 0.00001]. This finding suggests that the overall result of the electroacupuncture subgroup may have been influenced by a single study with a relatively large effect size. In Wang YN’s study, both the experimental and control groups received additional pharmacological treatment alongside conventional rehabilitation, which differed from the intervention protocols used in the other included studies. The combined use of drug therapy may have influenced the treatment effect and contributed to the observed heterogeneity.

The test for subgroup differences demonstrated a statistically significant difference between traditional acupuncture and electroacupuncture (*p* = 0.0004, *I^2^* = 92.2%), suggesting that the effects of acupuncture on EMG outcomes may vary according to acupuncture modality. Overall, the findings indicate that traditional acupuncture was superior to conventional treatment in improving muscle electrical activity. Although the initial pooled analysis did not demonstrate a significant advantage for electroacupuncture, the sensitivity analysis supported a beneficial effect of electroacupuncture on EMG outcomes, indicating that the result was relatively sensitive to individual studies and should therefore be interpreted with caution.

##### 3.1.4.5 BI

A total of 11 studies (8, 21, 27, 35, 50, 61–63, 67, 72, 73) reported the BI outcomes. The traditional acupuncture subgroup included seven studies and showed low heterogeneity among the included studies (*I^2^* = 10%, *p* = 0.36). A fixed-effects model was applied, and the pooled analysis demonstrated a significant improvement in BI compared with conventional treatment [MD = 7.86, 95% CI (6.84, 8.88), *p* < 0.00001] (Supplementary Figure S4). The electroacupuncture subgroup initially included three studies and demonstrated substantial heterogeneity (*I^2^* = 98%, *p* < 0.00001). Sensitivity analysis in the Supplementary Figure S5 identified the study by Meng et al. (35) as a major source of heterogeneity. After exclusion of this study, heterogeneity was completely eliminated (*I^2^* = 0%, *p* = 0.82). In addition to electroacupuncture, the experimental group in Meng CX’s study received functional electrical stimulation, which was not incorporated into the intervention protocols of the other included studies. This difference in the treatment regimen may have introduced clinical heterogeneity and contributed to the variability observed across studies. The pooled analysis based on the remaining two studies showed that electroacupuncture significantly improved BI compared with conventional treatment [MD = 14.85, 95% CI (12.63, 17.06), *p* < 0.00001], indicating a robust beneficial effect on activities of daily living. Only one study by Zhang et al. (50) involving special acupuncture reported BI. Therefore, quantitative synthesis for this subgroup was not performed. The test for subgroup differences indicated a significant difference between traditional acupuncture and electroacupuncture (*p* < 0.00001, *I^2^* = 96.8%), suggesting that the magnitude of improvement in BI varied according to acupuncture modality. Overall, both traditional acupuncture and electroacupuncture demonstrated superior efficacy compared with conventional treatment in improving activities of daily living, with electroacupuncture exhibiting a greater improvement in BI.

##### 3.1.4.6 BBS

A total of eight studies (30, 31, 37, 47, 48, 62, 63, 72) reported the BBS outcomes. The traditional acupuncture subgroup included four studies. The traditional acupuncture subgroup included four studies and demonstrated substantial heterogeneity (*I^2^* = 90%, *p* < 0.00001). A random-effects model was applied, and the pooled analysis showed that traditional acupuncture significantly improved BBS scores compared with conventional treatment [MD = 3.54, 95% CI (2.02, 5.06), *p* < 0.00001]. The electroacupuncture subgroup included three studies and showed low heterogeneity (*I^2^* = 21%, *p* = 0.28). A random-effects model was used, and the pooled results indicated that electroacupuncture significantly improved BBS scores compared with conventional treatment [MD = 5.39, 95% CI (3.74, 7.03), *p* < 0.00001] (Supplementary Figure S6). Only one study by Li et al. (48) reported BBS outcomes in the special acupuncture subgroup. Therefore, quantitative synthesis could not be performed for this subgroup. The test for subgroup differences was not statistically significant (*p* = 0.11, *I^2^* = 61.7%), suggesting that the effects of traditional acupuncture and electroacupuncture on balance function were generally comparable. Overall, both traditional acupuncture and electroacupuncture demonstrated superior efficacy compared with conventional treatment in improving balance performance after stroke, while current evidence remains insufficient to draw conclusions regarding special acupuncture.

##### 3.1.4.7 MAS

A total of seven studies (26, 27, 31, 44, 55, 58, 60) reported the MAS outcomes. The traditional acupuncture subgroup included two studies and demonstrated substantial heterogeneity (*I^2^* = 86%, *p* = 0.007). Therefore, a random-effects model was applied. The pooled analysis showed that traditional acupuncture significantly reduced MAS scores compared with conventional treatment [MD = −0.67, 95% CI (−1.19, −0.15), *p* = 0.01]. The electroacupuncture subgroup included four studies and showed moderate heterogeneity (*I^2^* = 44%, *p* = 0.15). A random-effects model was used, and the pooled results demonstrated a significant reduction in MAS scores following electroacupuncture treatment compared with conventional treatment [MD = −0.54, 95% CI (−0.76, −0.31), *p* < 0.00001] (Supplementary Figure S7). Only one study by Qian et al. (55) reported MAS outcomes in the special acupuncture subgroup; therefore, quantitative synthesis could not be performed for this subgroup. The test for subgroup differences was not statistically significant (*p* = 0.65, *I^2^* = 0%), indicating that the effects of traditional acupuncture and electroacupuncture on muscle spasticity were comparable. Overall, both traditional acupuncture and electroacupuncture were superior to conventional treatment in reducing post-stroke muscle spasticity, as reflected by lower MAS scores.

##### 3.1.4.8 Triceps surae muscle tone

A total of eight studies (3, 10, 45, 59, 64, 65, 69, 72) reported the triceps surae muscle tone outcomes. All studies reporting this outcome used traditional acupuncture, and no studies involving electroacupuncture or special acupuncture reported it. Therefore, only traditional acupuncture was analyzed for this indicator. The initial meta-analysis demonstrated substantial heterogeneity among the included studies (*I^2^* = 79%, *p* < 0.0001). Therefore, a random-effects model was applied. The pooled analysis demonstrated a significant reduction in triceps surae muscle tone following traditional acupuncture treatment compared with conventional treatment [MD = −0.43, 95% CI (−0.56, −0.29), *p* < 0.00001] (Supplementary Figure S8). The negative mean difference indicates lower muscle tone in the traditional acupuncture group.

Since no eligible studies involving electroacupuncture or special acupuncture were identified, subgroup comparisons between different acupuncture modalities could not be performed for this outcome. Although the pooled estimate favored traditional acupuncture, the substantial heterogeneity indicates variability in the magnitude of effect across studies.

##### 3.1.4.9 FAC

A total of five studies (11, 36, 45, 65, 66) reported the FAC outcomes. As all included studies assessed traditional acupuncture and no eligible studies involving electroacupuncture or special acupuncture reported this outcome, only the traditional acupuncture was included in the analysis. The initial meta-analysis demonstrated substantial heterogeneity among the included studies (*I^2^* = 74%, *p* = 0.003). Therefore, a random-effects model was applied. The pooled results showed that traditional acupuncture significantly improved FAC scores compared with conventional treatment [MD = 0.63, 95% CI (0.42, 0.85), *p* < 0.00001] (Supplementary Figure S9).

Sensitivity analysis identified Huang et al. (66) as a major source of heterogeneity. In Huang CL’s study, the control group received repetitive transcranial magnetic stimulation in addition to routine rehabilitation, which may have reduced the relative treatment difference between groups and contributed to the heterogeneity. After excluding this study, heterogeneity was completely eliminated (*I^2^* = 0%, *p* = 0.40). The pooled effect size remained statistically significant [MD = 0.73, 95% CI (0.61, 0.86), *p* < 0.00001] (Supplementary Figure S10), indicating that the results were robust and reliable. These findings suggest that traditional acupuncture is superior to conventional treatment in improving walking ability and functional ambulation after stroke.

##### 3.1.4.10 CR

A total of three studies (11, 30, 65) reported the CR outcomes. As all included studies assessed traditional acupuncture and no studies involving electroacupuncture or special acupuncture reported this outcome, only the traditional acupuncture was analyzed. The pooled analysis demonstrated that traditional acupuncture significantly reduced CR compared with conventional treatment [MD = −4.13, 95% CI (−4.89, −3.37), *p* < 0.00001]. No heterogeneity was observed among the included studies (*I^2^* = 0%, *p* = 0.78) (Supplementary Figure S11), indicating consistent effects across studies. A lower CR reflects reduced excessive co-contraction of antagonist muscles and improved neuromuscular coordination. These findings suggest that traditional acupuncture is superior to conventional treatment in improving muscle coordination and motor control after stroke.

#### 3.1.5 GRADE assessment of evidence certainty

The certainty of evidence ranged from moderate to low. Moderate-certainty evidence was found for clinical effective rate, MAS, and FAC, whereas low-certainty evidence was found for FMA, AROM, EMG, BI, BBS, triceps surae muscle tone, and CR. Details are presented in Supplementary Material 4.

### 3.2 Publication bias

#### 3.2.1 BI

Publication bias for the BI was assessed using funnel plots combined with Egger’s linear regression test, with a total of 11 studies included. Egger’s test returned a *p*-value of 0.146, indicating no significant publication bias was detected. Visual inspection showed that the funnel plot (Supplementary Figure S12) was not perfectly symmetrical, which was mainly attributable to the limited sample size; therefore, the study results are moderately credible.

#### 3.2.2 AROM

Fifteen studies were included in total. The funnel plot exhibited marked asymmetry, preliminarily suggesting the presence of publication bias. Egger’s test yielded a *p*-value of 0.013, which was less than 0.05. Therefore, the trim-and-fill method was applied for correction (Supplementary Figure S13), with seven hypothetical studies imputed, increasing the total number of studies to 22. After correction, the pooled effect sizes under both the fixed-effect and random-effects models decreased; nevertheless, the 95% CI of both models did not cross the line of no effect, and the effects remained statistically significant (*p* < 0.001). These findings suggest that the observed effect may be influenced by potential missing evidence or small-study effects. However, because this outcome-level analysis combined clinically distinct acupuncture modalities and showed substantial heterogeneity, the trim-and-fill results should be interpreted as exploratory and do not establish publication bias.

#### 3.2.3 EMG

Eleven studies were included in total. Visual inspection identified asymmetry in the funnel plot. Egger’s test produced a *p*-value of 0.013, indicating significant publication bias. After correction via the trim-and-fill method with five imputed studies, the total number of studies increased to 16 (Supplementary Figure S14). These findings suggest that the observed effect may be affected by potential missing evidence or small-study effects. Because substantial heterogeneity and clinically diverse acupuncture modalities were included in this outcome-level analysis, the adjusted results should be interpreted cautiously.

#### 3.2.4 FMA

In this study, publication bias analyses were conducted for the overall pooled dataset (37 studies, including conventional acupuncture, electroacupuncture, and special acupuncture techniques) and the conventional acupuncture subgroup (25 studies), respectively.

For the overall dataset, Egger’s test yielded a *p*-value of 0.007, confirming the presence of publication bias. After 14 hypothetical studies were imputed using the trim-and-fill method, the total sample size increased to 51 studies. The pooled effect size decreased slightly, whereas the 95% CI did not cross the line of no effect, and the result remained statistically significant (*p* < 0.001) (Supplementary Figure S15).

For the conventional acupuncture subgroup, Egger’s test returned a *p*-value of 0.033, also indicating publication bias. Ten hypothetical studies were imputed, resulting in a total of 35 studies. The corrected effect size declined, yet the statistical significance persisted (Supplementary Figure S16).

These findings demonstrate that significant publication bias existed in both the overall dataset and the conventional acupuncture subgroup. Since the overall direction of effect remained unchanged after adjustment, the conclusions are still robust.

#### 3.2.5 Clinical effective rate

Publication bias analyses were performed separately for the overall pooled studies (26 studies in total, including conventional acupuncture, electroacupuncture, and special acupuncture techniques) and the conventional acupuncture subgroup (16 studies) in the present study. The *p*-values from Egger’s test were *p* < 0.001 for both analyses, suggesting potential small-study effects (Supplementary Figure S17).

After 12 hypothetical studies were imputed via the trim-and-fill method, the number of overall studies increased to 38. The pooled effect size decreased markedly after adjustment, and the 95% CI crossed the line of no effect. For the conventional acupuncture subgroup, seven hypothetical studies were imputed, resulting in a total of 23 studies. The adjusted effect size declined, with its CI also crossing the line of no effect (Supplementary Figure S18).

These findings suggest that the observed benefit for clinical effective rate may have been substantially influenced by potential missing evidence or small-study effects.

In summary, Egger’s regression test did not detect statistically significant funnel-plot asymmetry for the BI. Exploratory analyses for AROM, EMG, FMA, and clinical effective rate suggested potential small-study effects. After trim-and-fill analyses, the pooled effects for AROM, EMG, and FMA remained statistically significant but were attenuated, whereas the adjusted estimates for clinical effective rate included the line of no effect.

### 3.3 Data mining analysis

#### 3.3.1 Acupuncture point frequency analysis

Among the 59 included studies, a total of 90 acupuncture points were identified, with a cumulative frequency of 395. Of these, 20 points were used at least five times, accounting for 70.89% of the total frequency. The top three most frequently used points were Yanglingquan (GB34) (37 times, 9.37%), Zusanli (ST36) (37 times, 9.37%), and Jiexi (ST41) (31 times, 7.85%). Acupuncture points used at least eight times are listed in Table 1.

**Table 1:** Frequency analysis of high-frequency acupuncture points in the management of post-stroke foot drop.

No.	Acupuncture point	Frequency/Times	Percentage/%
1	Yanglingquan (GB34)	37	9.37
2	Zusanli (ST36)	37	9.37
3	Jiexi (ST41)	31	7.85
4	Taichong (LR3)	22	5.57
5	Xuanzhong (GB39)	20	5.06
6	Zhaohai (KI6)	18	4.56
7	Sanyinjiao (SP6)	15	3.80
8	Qiuxu (GB40)	13	3.29
9	Zhongfeng (LR4)	12	3.04
10	Guangming (GB37)	9	2.28
11	Hegu (LI4)	9	2.28
12	Taixi (KI3)	8	2.03
13	Baihui (GV20)	7	1.77
14	Fenglong (ST40)	7	1.77
15	Huantiao (GB30)	7	1.77
16	Fengchi (GB20)	6	1.52
17	Shenmai (BL62)	6	1.52
18	Xuehai (SP10)	6	1.52
19	Quchi (LI11)	5	1.27
20	Chengshan (BL57)	5	1.27

#### 3.3.2 Association rule analysis

Using SPSS 18.0 statistical software, we conducted an association rule analysis on acupuncture points with a frequency of ≥5 times. The analysis was set with a minimum support of 30%, a minimum confidence of 80%, and a maximum antecedent count of 2. A total of 16 association rules were identified. The most prominent rules based on support included Jiexi (ST41) → Yanglingquan (GB34) (62%), Jiexi (ST41) and Zusanli (ST36) → Yanglingquan (GB34) (48%), and Taichong (LR3) → Zusanli (ST36) (42%). By constructing a network diagram of high-frequency acupuncture points, we found that thicker lines linking the points indicated a closer relationship between the points. The results are presented in Table 2 and Figure 5.

**Table 2:** Association rule analysis of high-frequency acupuncture points in the therapy of foot inversion after stroke.

Consequent	Antecedent	Support (%)	Confidence (%)
Yanglingquan (GB34)	Jiexi (ST41)	62	93.5483871
Yanglingquan (GB34)	Jiexi (ST41) and Zusanli (ST36)	48	95.83333333
Zusanli (ST36)	Taichong (LR3)	42	100
Yanglingquan (GB34)	Taichong (LR3)	42	80.95238095
Yanglingquan (GB34)	Taichong (LR3) and Zusanli (ST36)	42	80.95238095
Zusanli (ST36)	Xuanzhong (GB39)	40	90
Yanglingquan (GB34)	Xuanzhong (GB39)	40	80
Yanglingquan (GB34)	Zhaohai (KI6)	36	83.33333333
Jiexi (ST41)	Taichong (LR3) and Yanglingquan (GB34)	34	82.35294118
Zusanli (ST36)	Taichong (LR3) and Yanglingquan (GB34)	34	100
Jiexi (ST41)	Xuanzhong (GB39) and Yanglingquan (GB34)	32	87.5
Zusanli (ST36)	Xuanzhong (GB39) and Yanglingquan (GB34)	32	87.5
Zusanli (ST36)	Sanyinjiao (SP6)	30	80
Yanglingquan (GB34)	Sanyinjiao (SP6)	30	80
Zusanli (ST36)	Xuanzhong (GB39) and Jiexi (ST41)	30	86.66666667
Yanglingquan (GB34)	Xuanzhong (GB39) and Jiexi (ST41)	30	93.33333333

#### 3.3.3 Cluster analysis of high-frequency acupuncture points

Using SPSS 26.0 statistical software, we performed a cluster analysis on acupuncture points with a frequency of ≥5 times. The dendrogram revealed that the included acupuncture points were divided into three main clusters:

Cluster 1: Zhongfeng (LR4) → Guangming (GB37) → Sanyinjiao (SP6) → Yanglingquan (GB34) → Jiexi (ST41) → Zusanli (ST36) → Taichong (LR3) → Xuanzhong (GB39).
Cluster 2: Zhaohai (KI6) → Qiuxu (GB40) → Shenmai (BL62) → Chengshan (BL57) → Taixi (KI3).
Cluster 3: Baihui (GV20) → Fengchi (GB20) → Huantiao (GB30) → Hegu (LI4) → Quchi (LI11) → Fenglong (ST40) → Xuehai (SP10).

## 4 Discussion

In recent years, acupuncture has become increasingly common in clinical practice for treating foot drop, a sequela of stroke. Nevertheless, previous studies have generally been limited by relatively small sample sizes, variable intervention protocols, and incomplete methodological reporting. Previous evidence syntheses have also mainly evaluated electroacupuncture within broader electrical stimulation therapies rather than specifically examining acupuncture modalities and acupoint prescription patterns for post-stroke foot drop. Currently, foot drop after stroke is a complex motor impairment characterized by reduced ankle dorsiflexion, abnormal muscle tone, impaired neuromuscular coordination, and gait dysfunction. Although foot drop does not affect patients’ lifespans, its negative impacts on individual life, social quality of life, and psychological stress should be emphasized by clinicians and researchers. Conventional rehabilitation remains the foundation of treatment; however, residual ankle dysfunction and gait impairment remain common, highlighting the need for complementary rehabilitation approaches. Yan et al. (18) reported significant clinical efficacy by adding acupuncture at specific points (e.g., Hegu (LI4), Quchi (LI11), Futu (ST32), Zusanli (ST36), Shangjuxu (ST37), Xiajuxu (ST39), Fenglong (ST40), Jiexi (ST41), Neiting (ST44), and Yanglingquan (GB34)) on the affected side to the control group’s treatment. Similarly, Ke et al. (30) combined electroacupuncture at nine points (Yanglingquan (GB34), Jiexi (ST41), Zhongfeng (LR4), Taichong (LR3), Zusanli (ST36), Guangming (GB37), Xuanzhong (GB39), Sanyinjiao (SP6), and Zhaohai (KI6)) with the control group’s treatment and achieved related clinical efficacy. These studies also illustrate the diversity of acupuncture prescriptions and stimulation strategies used in clinical practice, which may partly explain variation in effect sizes across trials.

This study systematically included relevant clinical trials and evaluated the effects of acupuncture-based interventions and common acupoint prescription patterns for post-stroke foot drop. Given the differences in stimulation procedures, treatment parameters, and potential mechanisms among traditional acupuncture, electroacupuncture, and special acupuncture techniques, these interventions were interpreted through modality-specific analyses rather than as one fully homogeneous intervention. These categories should therefore be understood as pragmatic analytical strata rather than interchangeable intervention classes or direct comparative estimates of efficacy. In particular, the special acupuncture category was used to avoid pooling sparsely reported and procedurally distinct techniques, such as fire needle and scalp acupuncture, with conventional manual acupuncture or electroacupuncture; it does not support a class-level conclusion regarding the efficacy of any individual special technique. Overall, the present findings suggest that adding traditional acupuncture or electroacupuncture to conventional treatment may provide additional functional benefits for patients with post-stroke foot drop. However, owing to the high or unclear risk of bias in many included studies, substantial heterogeneity in several analyses, and potential small-study effects for selected outcomes, these findings should be interpreted cautiously.

The study results indicated that compared with conventional treatment alone, traditional acupuncture and electroacupuncture showed generally favorable effects across several clinically relevant outcomes. Both modalities were associated with improved FMA scores. Traditional acupuncture was also associated with favorable effects on AROM, EMG, BI, BBS, MAS, triceps surae muscle tone, FAC, and CR, whereas electroacupuncture showed favorable effects on AROM, BBS, and MAS. The evidence for special acupuncture techniques was comparatively limited because few studies were available for most outcomes.

For the FMA, traditional acupuncture and electroacupuncture were associated with pooled improvements of 6.25 and 7.99 points, respectively. These estimates were numerically comparable to, or exceeded, the 6-point minimal clinically important difference reported for the lower-extremity FMA in chronic stroke patients by Pandian et al. (74), supporting the potential clinical relevance of the observed motor improvements. This comparison should be interpreted together with the overall evidence profile, including heterogeneity and variation in intervention protocols.

For the BI, traditional acupuncture showed a significant pooled improvement, while the electroacupuncture result became favorable after exclusion of an influential study involving additional functional electrical stimulation. These findings suggest that acupuncture-based interventions may contribute to improved activities of daily living when used alongside conventional treatment.

For AROM, the results indicated that acupuncture intervention improved ankle mobility, with favorable effects observed in the traditional acupuncture, electroacupuncture, and special acupuncture subgroups. Both traditional acupuncture and electroacupuncture were also associated with lower MAS scores, suggesting potential benefits for reducing lower-limb spasticity.

Analyses of EMG, triceps surae muscle tone, CR, and BBS further showed generally favorable effects in relevant modality-specific analyses. Traditional acupuncture was associated with improved EMG values, reduced triceps surae muscle tone, and lower CR values, suggesting possible improvements in muscle activation, excessive co-contraction, and neuromuscular coordination. Both traditional acupuncture and electroacupuncture were associated with improved balance.

For clinical effective rate, acupuncture increased the effective rate by approximately 26% compared with conventional treatment alone. However, this outcome was variably defined across studies and may include subjective or composite criteria. Exploratory trim-and-fill analyses also suggested that the adjusted estimate crossed the line of no effect. Therefore, clinical effective rate should be regarded as a supportive outcome rather than the principal basis for conclusions regarding efficacy.

In summary, the modality-specific analyses showed a generally favorable and clinically coherent pattern across motor function, ankle mobility, spasticity, balance, gait-related function, and activities of daily living. This pattern supports the potential role of acupuncture-based interventions as adjunctive rehabilitation approaches for post-stroke foot drop.

Data-mining results revealed that the top three most frequently used acupuncture points were Yanglingquan, Zusanli, and Jiexi. The most prominent association rules included Yanglingquan–Jiexi, Yanglingquan–Jiexi–Zusanli, and Zusanli–Taichong. The cluster analysis also identified several recurrent acupoint combinations. These findings indicate that lower-limb acupoints related to ankle movement, lower-limb motor control, and gait rehabilitation were commonly incorporated into clinical prescriptions. However, acupoint frequency and co-occurrence should be regarded as descriptive and hypothesis-generating findings rather than evidence that one specific prescription is superior.

The included studies exhibited high substantial heterogeneity. First, clinical heterogeneity existed. The patient populations differed in terms of disease course, severity of illness, and traditional Chinese medicine syndromes. Meanwhile, the acupuncture intervention protocols were inconsistent, with substantial variations in acupoint selection, needling techniques, treatment frequency, and duration of treatment. In addition, the assessment time points and diagnostic criteria for different outcome measures were not fully consistent, which was a major contributor to the increased heterogeneity. Second, methodological heterogeneity was observed. The sample sizes of the included studies varied, and differences existed in the methodological quality of randomization, blinding, and other designs among some studies. Variations in data measurement and statistical methods further exacerbated the heterogeneity. Finally, heterogeneity was also affected by therapeutic and regional characteristics. All studies included in this research were conducted in China, mainly because acupuncture therapy has strong regional characteristics and is more widely applied in clinical practice and related scientific research in China. During the literature screening and inclusion process, we performed a comprehensive search and verification of relevant studies worldwide and confirmed that no eligible studies from other countries met the inclusion criteria. Therefore, heterogeneity indicates that the magnitude of benefit may vary across clinical settings and treatment protocols, rather than necessarily contradicting the generally favorable direction of effect observed across several outcomes. Although modality-specific subgroup analyses were conducted to reduce inappropriate pooling of distinct acupuncture interventions, residual heterogeneity remained in several analyses. Accordingly, the pooled estimates should be interpreted as an overall treatment signal rather than a precise effect applicable to every patient or acupuncture prescription.

To further evaluate the reliability of these findings, we assessed the certainty of evidence using the GRADE approach. The GRADE assessment indicated that the certainty of evidence ranged from moderate to low. Moderate-certainty evidence supported the effects of acupuncture on clinical effective rate, MAS, and FAC, whereas low-certainty evidence suggested potential benefits for FMA, AROM, EMG, BI, BBS, triceps surae muscle tone, and CR. These findings suggest that acupuncture may be beneficial for improving motor function, walking ability, muscle spasticity, balance, and activities of daily living in patients with post-stroke foot drop. However, the certainty of evidence was limited mainly by methodological limitations, heterogeneity, imprecision, and potential publication bias. Therefore, these findings should be interpreted in the context of the methodological limitations of the included studies, and further well-designed randomized controlled trials are warranted to strengthen the evidence base. In addition, adverse events were insufficiently reported across the included studies. Therefore, although acupuncture-based interventions showed potentially favorable functional effects, this review could not reliably assess their safety for post-stroke foot drop. Future trials should systematically collect and transparently report adverse events, including their type, severity, timing, and potential relationship to treatment.

The principal limitation of this review is the serious methodological risk of bias in the included trials. Allocation concealment was unclear in 58 of the 59 studies, and the risk of bias related to blinding was high in all included studies. These limitations are particularly relevant because several outcomes, including motor function, balance, walking ability, and clinical effective rate, may be influenced by participant expectations, treatment delivery, or outcome assessment. Consequently, the observed pooled effects may overestimate the true magnitude of benefit. Although subgroup and sensitivity analyses were conducted to explore statistical heterogeneity and the influence of individual studies, these analyses cannot eliminate systematic bias originating from the primary trials. Therefore, the present findings should be interpreted as indicating possible additional benefits of acupuncture-based interventions rather than providing definitive evidence of efficacy. Future trials should use adequately concealed allocation, prospectively registered protocols, blinded outcome assessment where feasible, and transparent reporting of all prespecified outcomes.

It is worth noting that some articles introduced potential confounding factors in their interventions. These studies may not accurately isolate the effects of acupuncture therapy, making it difficult to draw definitive conclusions about its efficacy in treating foot drop after stroke. Future studies should strictly adhere to the single-variable principle to explore the clinical efficacy of acupuncture as an independent therapy, providing stronger evidence.

To further validate the results of this study, it is necessary to follow the reporting standards of acupuncture clinical practice guidelines (75, 76), focus on the implementation of blinding (75), and conduct multicenter comparative trials with larger sample sizes and smaller study biases. These studies will provide more reliable clinical evidence for acupuncture in treating foot drop after stroke. They will guide researchers and clinicians in related fields to have a new perspective on studying or treating foot drop after stroke, helping them make wiser decisions, promoting the development of acupuncture, and providing effective solutions to alleviate the suffering of patients with foot drop (78). Taken together, acupuncture-based interventions may be associated with improved functional outcomes in post-stroke foot drop; however, the serious risk of bias across the included trials means that the magnitude and certainty of these effects remain uncertain. Definitive conclusions regarding efficacy, safety, or the relative effectiveness of different acupuncture modalities require larger, prospectively registered, rigorously conducted, and transparently reported randomized controlled trials.

## Data Availability

The original contributions presented in the study are included in the article/Supplementary material, further inquiries can be directed to the corresponding authors.
